# Supplementary material for: Time-series transcriptome analysis identified differentially expressed genes in broiler chicken infected with mixed Eimeria species
Source: Front Genet. 2022 Aug 8;13:886781. doi: 10.3389/fgene.2022.886781 (PMC9393255; doi:10.3389/fgene.2022.886781)
Supplement: Supplementary file 2 [file DataSheet1.ZIP › 4dpi_KEGG.Gsea.1625100298584/KEGG_DNA_REPLICATION.html]

Details for gene set KEGG\_DNA\_REPLICATION[GSEA]

|  || Dataset | TMM\_4dpi\_gct\_format\_4dpi\_gct\_format.Class\_4dpi.cls #PC\_versus\_NC.Class\_4dpi.cls #PC\_versus\_NC\_repos |
| Phenotype | Class\_4dpi.cls#PC\_versus\_NC\_repos |
| Upregulated in class | 1 |
| GeneSet | KEGG\_DNA\_REPLICATION |
| Enrichment Score (ES) | 0.44621742 |
| Normalized Enrichment Score (NES) | 1.3801402 |
| Nominal p-value | 0.08095741 |
| FDR q-value | 0.48016068 |
| FWER p-Value | 0.9996 |
Table: GSEA Results Summary

  

Fig 1: Enrichment plot: KEGG\_DNA\_REPLICATION      
 Profile of the Running ES Score & Positions of GeneSet Members on the Rank Ordered List

  

| SYMBOL | TITLE | RANK IN GENE LIST | RANK METRIC SCORE | RUNNING ES | CORE ENRICHMENT || 1 | RPA3 | na | 637 | 0.856 | 0.0309 | Yes |
| 2 | RFC3 | na | 848 | 0.740 | 0.0859 | Yes |
| 3 | MCM6 | na | 1454 | 0.532 | 0.0876 | Yes |
| 4 | MCM3 | na | 1557 | 0.506 | 0.1287 | Yes |
| 5 | LIG1 | na | 1562 | 0.504 | 0.1779 | Yes |
| 6 | POLE | na | 1769 | 0.462 | 0.2060 | Yes |
| 7 | MCM4 | na | 1815 | 0.452 | 0.2466 | Yes |
| 8 | RNASEH2B | na | 1911 | 0.437 | 0.2816 | Yes |
| 9 | PRIM2 | na | 2174 | 0.393 | 0.2983 | Yes |
| 10 | MCM5 | na | 2220 | 0.386 | 0.3323 | Yes |
| 11 | DNA2 | na | 2225 | 0.385 | 0.3698 | Yes |
| 12 | MCM2 | na | 2631 | 0.331 | 0.3685 | Yes |
| 13 | RFC1 | na | 2770 | 0.312 | 0.3876 | Yes |
| 14 | FEN1 | na | 2781 | 0.311 | 0.4172 | Yes |
| 15 | RFC4 | na | 2926 | 0.292 | 0.4338 | Yes |
| 16 | POLE2 | na | 3146 | 0.263 | 0.4414 | Yes |
| 17 | RPA2 | na | 3366 | 0.236 | 0.4462 | Yes |
| 18 | POLA1 | na | 3695 | 0.199 | 0.4383 | No |
| 19 | RPA1 | na | 4548 | 0.120 | 0.3790 | No |
| 20 | PRIM1 | na | 4619 | 0.114 | 0.3843 | No |
| 21 | RFC2 | na | 5053 | 0.074 | 0.3554 | No |
| 22 | PCNA | na | 5126 | 0.068 | 0.3561 | No |
| 23 | POLE3 | na | 5405 | 0.045 | 0.3373 | No |
| 24 | POLD2 | na | 5963 | -0.003 | 0.2912 | No |
| 25 | RNASEH1 | na | 6603 | -0.055 | 0.2432 | No |
| 26 | RFC5 | na | 8163 | -0.194 | 0.1321 | No |
| 27 | SSBP1 | na | 9368 | -0.331 | 0.0641 | No |
| 28 | POLD3 | na | 11081 | -0.631 | -0.0169 | No |
| 29 | POLE4 | na | 11699 | -0.962 | 0.0260 | No |
Table: GSEA details [plain text format]

  

Fig 2: KEGG\_DNA\_REPLICATION      
 Blue-Pink O' Gram in the Space of the Analyzed GeneSet

  

Fig 3: KEGG\_DNA\_REPLICATION: Random ES distribution      
 Gene set null distribution of ES for **KEGG\_DNA\_REPLICATION**
